# Introduction to the Special Issue on Computer Science Education

**DOI:** 10.1007/s11528-022-00741-w

**Published:** 2022-04-22

**Authors:** Cornelia Connolly, Tony Hall

**Affiliations:** 1 School of Education, National University of Ireland, Galway, Galway, Ireland; 2 Lero, SFI Centre for Software Research, National University of Ireland Galway, Galway, Ireland

Computing is pervasive in society, enterprise and education, underpinning all aspects of our lives today. Indeed, it would be a singular achievement to identify some part of contemporary life not supported, in some way, by technology. Furthermore, learning, teaching and assessment have become increasingly mediated by computing and technology, and especially during the global COVID-19 pandemic.

Computer Science (or subjects relating to this discipline) offers young people the opportunity to move away from being passive users of computing to becoming designers of computer systems and applications.

As a result of the increasing ubiquity of computing in our world, the importance of supporting creative and quality education in Computer Science (CS) is incontrovertibly an educational imperative for the world of today, and tomorrow. Indeed, agreement on the definition of Computer Science is the first challenge. For our purpose, Computer Science is understood as a scientific discipline that covers 'principles such as algorithms, data structures, programming, systems architecture, design, problem-solving etc.;' (The Royal Society & Furber, 2012, p. 17) Computer Science education (CSE) has experienced unprecedented growth globally, particularly in the last decade, in terms of new initiatives, programmes, technologies and curricula to support learners to engage in CS, both in schools and outside: in not-for-profit and community-based, informal CS learning initiatives.

For example, in the editors’ own context of Ireland, Computer Science has been introduced for the first time as an assessable and examinable subject for high-stakes college entry, the Leaving Certificate—with CS accorded full status and parity of esteem on the Irish second-level school curriculum, alongside the traditional, core subjects, e.g. Mathematics, English, Irish etc.

This has resulted also in the growth and development of CS education for teachers. Both editors, with the first editor as lead, were centrally involved in the design, development and accreditation of Ireland’s first undergraduate (concurrent) teacher education degree programme in CS: the BA Education (Computer Science and Mathematical Studies) at the National University of Ireland, Galway.

In 2024, this innovative teacher education programme will graduate Ireland’s first cohort of teachers who have completed a 4-year honours, denominated undergraduate teacher education degree to teach the Computer Science specification.

The remarkable growth of CS education internationally is to be warmly welcomed, yet at the same time it raises several important questions for all those passionately interested in computing and technology, and moreover the education of young people to think critically, and engage creatively with CS in school and beyond: throughout their lives as developing adults and productive citizens of the world.

What are the big issues in CS education now, for scholars, teachers, parents, students, policymakers, as the domain continues to develop internationally?

What supports, technologies and infrastructure do we need, to ensure the effective integration of CS in schools and beyond, e.g. in informal and non-formal domains and settings?

How do we ensure that CS education is inclusive, for all learners and young people globally?

How do we ensure that learners feel a sense of belonging, and that they belong to a diverse, global CS community?

The purpose of this special issue, a landmark first imprint of *TechTrends* on the topic of Computer Science Education, is to help the international research community to answer the aforesaid, salient questions. Contributing to the field and identifying the current state-of-the-art research in CS education internationally.

We are delighted to edit and present this special issue of *TechTrends* on CS education, including 13 papers presenting a wide variety of core challenges and requirements for the successful design, introduction and continued growth and development of CS education around the world. The papers range from inclusive design of CS education internationally to the foundational role of computational thinking, not only in promoting CS in schools, but in the critical development of general problem solving, across disciplines. We highlight here some of the many valuable and impactful contributions to this landmark issue.

Ensuring CS is taught by suitably, appropriately qualified teachers remains a key priority for educational jurisdictions and systems internationally. The paper by Koressel and colleagues highlights licensure of CS teachers, looking at Indiana (US) as a context for teacher preparation. This work underscores how CS education necessitates a *two-pronged approach*, where new CS teachers are trained from the existing in-service teaching population while also designing and deploying effective teacher education programmes that will suitably and effectively prepare the next generations of teachers ab initio in CS education.

Diversity and inclusion remain central to the development of CS education internationally, helping to ensure a sense of belonging for both students and teachers. Prevailing stereotypes and entrenched perspectives need to challenged and changed. The paper by Hoffman and colleagues provides a fascinating, compelling insight into culturally-relevant computing in the multicultural context of Hawaii. The authors undertook focus group interviews with in-service CS teachers; and critically, the paper contributes important findings with respect to culturally-relevant computing, and useful implications for designers, practitioners, and researchers working in CS education.

COVID-19 has highlighted the precariousness of our world, including the importance of computing and technology to enable us to remain connected, especially when we could not safely co-locate and be together in close proximity in the same physical space. As more and more learners engage with CS in schools and beyond, this necessitates further, increased professional development for CS educators and teachers. While we look with hope to a future post-pandemic, it does not preclude the chance that this may happen again and the need to pivot significantly to online learning and teaching. The article by Mouza and colleagues outlines how they shifted to a virtual professional development (PD) institute during COVID-19. The paper provides key insights for research and practice, highlighting how the design of the virtual PD institute resulted in increased teachers’ self-knowledge and efficacy, while also exemplifying the affordances of the virtual institute the teachers most valued. This paper makes for especially relevant and interesting reading for those interested in online/blended, hybrid and hyflex teacher PD.

Crucially, in their paper on computational thinking (CT), Yadav and colleagues look at the contribution that CT can make to metacognition as a core fundament of all learning. Traditionally, as Yadav and colleagues rightly point out, CT has been used as a mediating construct to introduce CS into K-12 education. Their paper in this special issue makes for fascinating reading with respect to how CT can be used to teach general problem-solving, as a critical basis for all learning across disciplines, e.g. the potential of abstraction in supporting the learning of mathematics. This paper makes a foundational contribution to deepen our understanding of how CT can be used to improve learning across disciplines, alongside its already well-established potential to support the integration of CS within school curricula globally.

Crucial to ensuring that CS education develops and grows in a foundationally inclusive way, where diversity is represented throughout the design of CS education will be to empower underrepresented groups to engage with Computer Science. The article by Ren makes a highly significant contribution to the special issue, drawing our attention to the underrepresentation of students in CS, especially women and women of color; the perpetuation of stereotypes, biases and oppressions; and the imperative to challenge these, and ensure CS is truly diverse and inclusive. Adopting Feminist pedagogy in CS, this paper highlights how *access does not mean success* in terms of the engagement of underrepresented students in CS, and the need to actively, critically change this. Through feminist consciousness, collaborative learning, and reflexive learning, Ren highlights key design considerations for truly inclusive CS.

This special issue makes a significant contribution to the international research literature. We thank all our authors, both those published here and also the authors who submitted proposals. We also take the opportunity to thank the Editor-In-Chief, Professor Charles B. Hodges and team of reviewers.

This special issue serves as an important touchstone publication, helping us to design the future of Computer Science Education globally—a future that is foundationally characterized by a critical and constructive approach to technology; meaningful, effective professional development for all CS teachers; and, most importantly, inclusion for all learners.

## References

[CR1] Furber S (2012). Shut down or restart? The way forward for computing in UK Schools.

